# *In-vivo* probabilistic atlas of human thalamic nuclei based on diffusion- weighted magnetic resonance imaging

**DOI:** 10.1038/sdata.2018.270

**Published:** 2018-11-27

**Authors:** Elena Najdenovska, Yasser Alemán-Gómez, Giovanni Battistella, Maxime Descoteaux, Patric Hagmann, Sebastien Jacquemont, Philippe Maeder, Jean-Philippe Thiran, Eleonora Fornari, Meritxell Bach Cuadra

**Affiliations:** 1 Department of Radiology, Centre Hospitalier Universitaire Vaudois (CHUV) and University of Lausanne (UNIL), Lausanne, Switzerland; 2 Centre d’Imagerie BioMédicale (CIBM), Lausanne University Hospital (CHUV) and University of Lausanne (UNIL), Lausanne, Switzerland; 3 Service of General Psychiatry, Department of Psychiatry, Centre Hospitalier Universitaire Vaudois (CHUV) and University of Lausanne (UNIL), Lausanne, Switzerland; 4 Memory and Aging Center, Department of Neurology, University of California, San Francisco, USA; 5 Sherbrooke Connectivity Imaging Lab (SCIL), Computer Science Department, Universite de Sherbrooke, Sherbrooke, Canada; 6 Department of Pediatrics, University Hospital Center Sainte-Justine, Montreal H3T 1C5, Canada; 7 Signal Processing Laboratory (LTS5), École Polytechnique Fédérale de Lausanne (EPFL), Lausanne, Switzerland

**Keywords:** Brain imaging, Magnetic resonance imaging, Brain

## Abstract

The thalamic nuclei are involved in many neurodegenerative diseases and therefore, their identification is of key importance in numerous clinical treatments. Automated segmentation of thalamic subparts is currently achieved by exploring diffusion-weighted magnetic resonance imaging (DW-MRI), but in absence of such data, atlas-based segmentation can be used as an alternative. Currently, there is a limited number of available digital atlases of the thalamus. Moreover, all atlases are created using a few subjects only, thus are prone to errors due to the inter-subject variability of the thalamic morphology. In this work, we present a probabilistic atlas of anatomical subparts of the thalamus built upon a relatively large dataset where the individual thalamic parcellation was done by employing a recently proposed automatic diffusion-based clustering method. Our analyses, comparing the segmentation performance between the atlas-based and the clustering method, demonstrate the ability of the provided atlas to substitute the automated diffusion-based subdivision in the individual space when the DW-MRI is not available.

## Background & Summary

The thalamus is a central relay structure and regulator of the motor and sensory impulses that are transmitted by the white matter fibers. Despite its relatively small size of approximatively 8*cm*^3^ per hemisphere, the thalamus represents a highly complex structure built from numerous small nuclei differing between them in both histology and function. Each nucleus projects to different parts of the cortex and therefore, is associated with different neurological function. For this reason, the thalamic nuclei are implicated in a wide range of functional impairments and, as consequence, are of central interest in many neurodegenerative studies and clinical applications^1–4^.

Magnetic Resonance Imaging (MRI), as a non-invasive technique that provides an enhanced depiction of the soft biological tissues, allows an automated delineation and characterization of the brain structures^5^. However, the intrinsic contrast provided by the standard T1- and T2-weighted (T1w and T2w) MRI is too low for distinguishing any thalamic subpart and therefore, these images are of limited use for segmenting the nuclei in an automated framework.

Diffusion-weighted MRI (DW-MRI) is, so far, the only non-invasive imaging method able to image the human brain white matter by describing the geometry of its underlying microstructure. More precisely, DW-MRI captures the average diffusion of water molecules, which probes the structure of the biological tissue at a scale much smaller than the imaging resolution. Hence, DW-MRI is able to depict the different fibers characteristics representing each thalamic nucleus with respect to its cortical projections. Therefore, the state-of-the-art methods in the field of automated thalamic parcellation are mainly based on features provided by DW-MRI and are divided in two groups: the first exploring the local diffusion properties^6–8^, while the second, the global thalamic connectivity^9,10^. The major difference between the groups in terms of outcome is that the fiber tracking approaches lead to a segmentation pattern that strongly diverges from the known anatomy of the thalamus^9,10^.

Recent literature proposes a robust and reproducible method that subdivides the thalamus in seven regions closely matching the anatomy^11^. The developed segmentation framework is based on the trade-off between the spatial position of the thalamic voxels and their local diffusion properties from 3 T MRI expressed by the diffusion orientation distribution function (ODF) projected in the spherical harmonics (SH) basis^12–14^. Unlike other DW-MRI features used so far, ODF allows the use of the full diffusion signal and orientation profile at each voxel and therefore, the proposed method outperforms the state-of-the-art based on exploring the local diffusion properties.

Nevertheless, if the DW-MRI data is not available, it would not be possible to perform such parcellation. One alternative in such cases is the use of digital atlases. Currently, there is a limited number of published works presenting thalamic atlases^9,15–18^, especially available in digital format^9,17,19^. In general, these atlases are built using a few subjects only and therefore, they enclose a restrain inter-subject variability of the thalamic morphology. Additionally, while Morel’s^16^ and Yelnik’s^17^ atlases are portraying the histology, Behrens’^9^ one subdivides the thalamus with respect to its cortical connections, which does not necessarily reflect the underlying anatomy.

**Figure 1:**
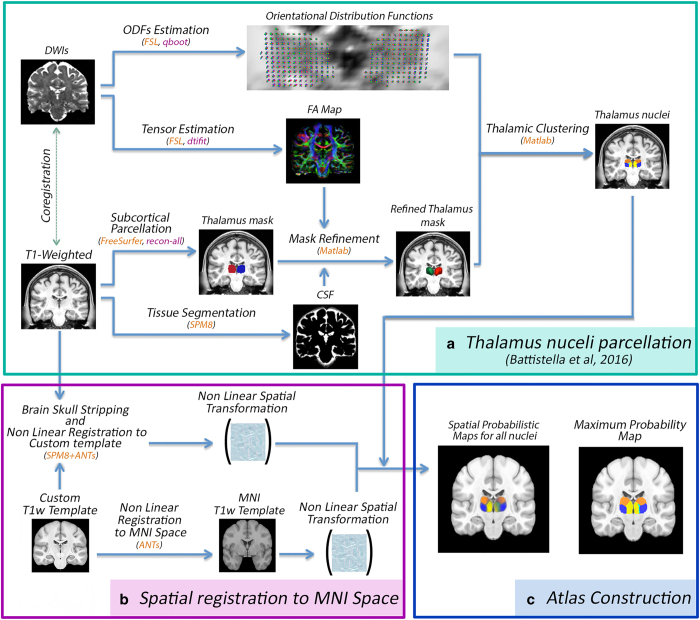
Processing flowchart showing the main steps for constructing the thalamic nuclei probabilistic atlas.

The aim of the present work is to provide a digital imaging-based probabilistic atlas of the anatomical thalamic subparts built from a relatively large population of healthy controls. To this end, we first segmented a considerably big subset of the Human Connectome Project data using previously proposed ODF-based thalamic clustering. From the obtained parcellations, we then constructed the atlas of each nuclei and finally, by calculating the respective mutual template, we transformed the outcome to the MNI (Montreal Neurological Institute) space (see Fig. 1).

Moreover, the atlas was compared with the ODF-based clustering in terms of parcellation outcome in a large testing set of healthy subjects. Our analysis shows highly similar segmentation patterns between both atlas-based and the cluster-based approaches, which demonstrates the ability of the provided atlas to substitute the automated ODF-based subdivision in the individual space when the DW-MRI is not available.

## Methods

In order to create a spatial probabilistic atlas map (SPAM) for fourteen thalamic nuclei, seven per hemisphere, a label-based approach^20^ was performed. First, for each subject, the right and the left thalami were automatically segmented into seven different nuclei each. Second, each T1w image was registered to the MNI space and the obtained spatial transformation was then applied to map the individual thalamic parcellation into a stereotaxic space. Finally, a probability map was constructed for each thalamic nucleus, by determining the proportion of subjects assigned to a given anatomic label at each voxel position, in stereotaxic space^20^. The flow chart in Fig. 1 illustrates the entire processing pipeline.

### Participants

Seventy unrelated healthy subjects (age range 22-36 years, 39 females) were selected from the Human Connectome Project (HCP) database. The choice of using this data is related to its high quality and better spatial resolution, which allowed an easier implementation of the designed procedures.

All subjects were scanned on the same 3 T Siemens Skyra platform with a 32 channels head coil at Washington University (WashU). The scanning protocol includes five MRI modalities, but only the T1w, the T2w and the DW-MRI were used in the present study.

***T1w***: Magnetization-Prepared Rapid Acquisition Gradient Echo (MPRAGE) sagittal images were acquired using a 3D inversion recovery sequence with echo time (TE) = 2.14 ms, repetition time (TR) = 2400 ms, inversion time (IT) = 1000 ms, flip angle (FA) = 8^o^, Bandwidth (BW) = 210 Hz per pixel, echo spacing (ES) = 7.6 ms, gradient strength = 42 mT/m, field of view (FOV) = 180 × 224 × 224 mm^3^, voxel size = 0.7 × 0.7 × 0.7 mm^3^ and acquisition time 7 min 40 s.

***T2w***: T2w sagittal images were acquired using a 3D T2 sampling perfection with application-optimized contrast by using flip angle evolution (SPACE) sequence with echo time (TE) = 565 ms, repetition time (TR) = 3200 ms, Bandwidth (BW) = 744 Hz per pixel, echo spacing (ES) = 3.53 ms, turbo factor = 314, field of view (FOV) = 180 × 224 × 224 mm^3^, voxel size = 0.7 × 0.7 × 0.7 mm^3^ and acquisition time 8 min 24 s.

***DW-MRI***: Multi-slice echo planar imaging (EPI) with multi-band (MB) excitation and multiple receivers were acquired with echo time (TE) = 289.5 ms, repetition time (TR) = 5520 ms, flip angle (FA) = 78^o^, refocusing flip angle (rFA) = 160^o^, Bandwidth (BW) = 1488 Hz per pixel, multiband factor = 3, echo spacing (ES) = 0.78 ms, gradient strength = 100 mT/m, field of view (FOV) = 210 × 180 × 138 mm^3^, voxel size = 1.25 × 1.25 × 1.25 mm^3^ and b-values = 0, 1000, 2000 and 3000 s/mm^2^. Each gradient table includes approximately 90 diffusion–weighted directions plus 6 b0 acquisitions interspersed each run. The acquisition time was around 63 min.

In addition to the raw MR images, the HCP database also provided the processed results (brain mask, cortical surfaces, cortical maps and volumetric/surface parcellations, etc.) obtained by a customized FreeSurfer pipeline, specially designed for this project. For more details about the acquisition protocol or processing pipeline see^21–23^.

### Thalamic parcellation

For the thalamic parcellation we followed the framework proposed by Battistella *et al.*^11^. Bellow we briefly summarize the main processing steps.

### Refinement of the thalamic mask

The first step consists in obtaining an accurate mask of the whole thalamus (see Fig. 1a).

In a nutshell, the methodology is as follows. First, each individual skull stripped T1w image was segmented in three different classes: the grey matter (GM), the white matter (WM) and the cerebro-spinal fluid (CSF), applying the modified Gaussian Mixture Model included in the SPM8 suite^24^. Using this approach, the probability of each voxel to belong to GM, WM or CSF was computed. Second, the DW-MRI acquired with b-value close to 1000 s/mm^2^ were employed to fit a diffusion tensor in order to obtain the fractional anisotropy map (FA). Third, all the thalamic masks, obtained from the Freesurfer subcortical parcellation, were refined to avoid partial volume contaminations. More precisely, on one hand we have eliminated voxels that had higher probability than 5% to represent the CSF and on the other, those that are more likely to be part of the internal capsule instead of the thalamus i.e. with FA values higher than 0.55 - a threshold that was empirically established. All these steps were performed in subject space.

### Thalamic nuclei clustering

We first compute the orientation distribution functions (ODFs) of the thalamus from high angular resolution diffusion images (HARDI) using q-Ball imaging with the constant solid angle algorithm (Qball-CSA)^25^. The Qball-CSA is included in the FSL (https://fsl.fmrib.ox.ac.uk/fsl/fslwiki/FDT/UserGuide) image processing suite throughout the Qboot function. The Qball-CSA algorithm was applied by setting the maximum number of ODF peaks to be detected to 2 using 50 samples for residual bootstrapping^1^, as in the default settings of the Qboot command in FSL. Moreover, in order to have a compatible ODF estimation model with a one-shell DWI data, as usually employed setting for this thalamic parcellation, we used the mono-exponential modeling considering the average signal from the three shells of the DWI data. The resulting ODFs from Qboot algorithm were represented in a basis of spherical harmonic (SH) functions^12,13^. The maximum SH basis order was set to 6 giving 28 SH coefficients in total. Thereby, the mean coefficients that were used for the clustering algorithm were estimated as the average of the samples of the ODF shapes in each voxel resulting from the Qboot bootstrapping.

The nuclei parcellation was performed by a modified k-means algorithm taking as inputs the mean ODF coefficients in SH basis and the position coordinates of each voxel from the final thalamic mask. The decision metric represents a weighted linear combination of Euclidean distances between the spatial position and the ODF coefficients for each voxel, respectively. The distance between the ODF coefficients will group voxels with similar microstructural properties, while the spatial distance, as proven empirically, will constrain them to be contiguous. Therefore, the spatial term will assure compact clusters in the final parcellation. An equal contribution was considered from both features, i.e. the weighting factor for each Euclidean distance was set to 0.5. The number of clusters was set to seven^11^. Moreover, as the two used features are not at the same scale of values, in order to avoid any bias in the k-means clustering, we have multiplied the ODF coefficients by a scaling factor that bring the Euclidean distances between them in the same range of values as the spatial distances. Hence, empirically we have set this scaling factor to 100. Additionally, to exclude an initialization bias, the method was initialized in a data-driven fashion by taking as centroids the average of 5000 randomly initialized k-means using only the position as the input feature.

**Figure 2:**
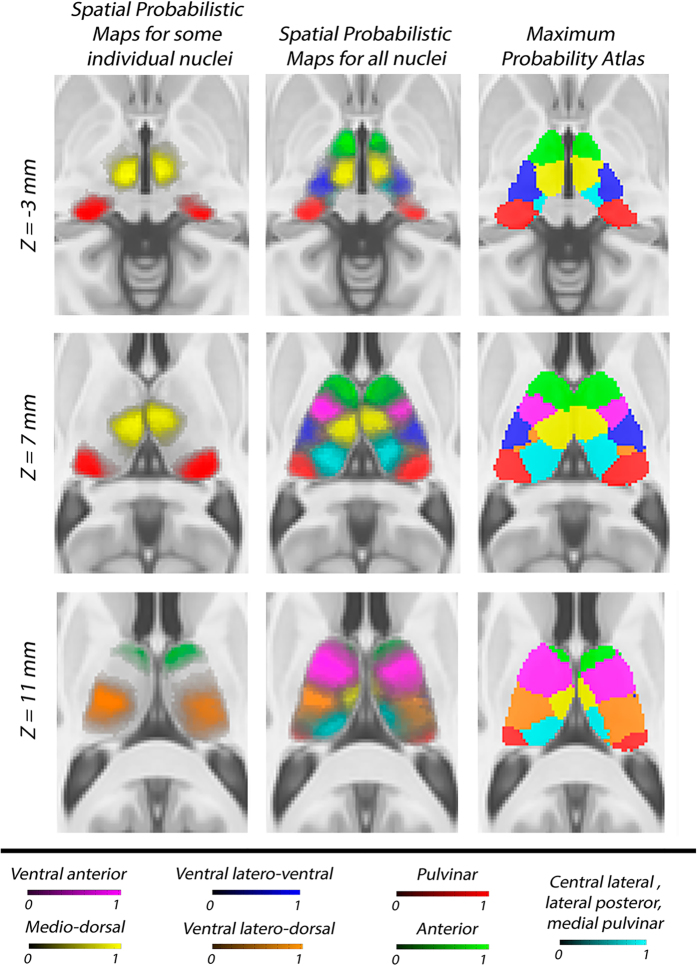
Thalamic nuclei atlas. Each row shows different slices of the T1152 template in MNI space focused on the thalamic area and overlaid with the spatial probabilistic maps (first and second column) and the maximum probability atlas (third column).

The final seven partitions derived from the clustering algorithm in each individual thalamus were labeled accordingly to the anatomical subpart they represent respectively. Namely, we have: anterior group (A), ventral anterior group (VA), medio-dorsal group (MD), ventral latero-ventral group (VLV),ventral latero-dorsal group (VLD), pulvinar (Pu) and cluster enclosing the central lateral, the lateral posterior and the medial pulvinar (CL-LP-PuM) (see Fig. 2).

For three out of the 70 tested cases, we observed a clustering outcome differing from the expected segmentation pattern, therefore they were not considered for the construction of the atlas.

### Custom template construction and spatial normalization

All the skull stripped T1w and T2w images were employed to build a customized template using the symmetric group-wise diffeomorphic normalization (SyN) method incorporated in the open source software Advanced Normalization Tools (ANTs)^26,27^. ANTs registration suite was used because it was selected as the top ranked registration procedure in terms of accuracy and precision in the recent nonlinear registration evaluation paper of Klein *et al.*^28^.

ANTs robustly maps images from a population to a common space by finding the template and the set of transformations that gives the “smallest” parameterization of the dataset. First, the T1w and T2w images were independently affine registered and averaged to create a first iteration template for both modalities. Second, all the T1w and T2w images were registered to their corresponding template using cross-correlation as similarity metric. Parameters were specified so the SyN registration method uses 30 iterations at a quarter resolution, 20 iterations at half resolution, and 4 iterations at full resolution. In the next step the aligned images were averaged to create new templates. The averaged volumes are then used as new templates for another iteration of the same procedure. This process was repeated six times and both a T1w and a T2w templates were obtained and the spatial transformations between subject space and custom atlas space (native-to-custom transformations) were saved.

The resulting custom T1w template was finally non linearly registered to MNI space using as target template the nonlinear version of the ICBM152 atlas introduced by Evans^29,30^. The spatial transformation (custom-to-MNI) between both templates was also saved.

More details about the SyN method can be found in^26^.

### Atlas construction

The probabilistic atlas for each thalamic nuclei was built following the strategy schematically presented in Fig. 1b and c. We applied the native-to-custom and custom-to-MNI transforms to resample the subject individual thalamic parcellation data into the MNI space. Since we were averaging across the subjects, for forming images with labels we applied nearest-neighbor as interpolation method. By averaging across the subjects, we then computed the probability maps for the 14 thalamic subparts (seven for each hemisphere). The maximum likelihood labeling was determined by identifying, at each voxel, the structure label with the greatest value in the nuclei probability maps. In the case of multiple modes, where multiple nuclei had maximal likelihood values at a voxel, a label was chosen at random from the modes at that site (see Fig. 2).

### Code availability

The custom code used for the thalamic nuclei clustering is implemented in Matlab and is uploaded in Zenodo (Data Citation 1). The used and the current version of the software is 1.0. All the parameters employed to process the dataset are given in the provided files.

## Data Records

A summary of the data records related to this study is given in Table 1.

### Data Records as a contribution

The contribution of the presented work is a single data record containing two image files given in compressed NIfTI-1 format (.nii.gz extension). One of them is a 4D volumetric file including all 14 constructed 3D spatial probabilistic maps of the thalamic nuclei, while the second one is 3D volume representing the maximum likelihood atlas where each voxel contains a single integer value for each thalamic nucleus.

The maximum likelihood atlas is supplied with a standard color lookup table (LUT, stored as a .txt file), where each line represents one labeled thalamic region and provides information about the respective label-code, name, RGB (red, green, blue) color triplet and opacity. The RGB and opacity values are 8-bits integers values. For visualization purposes, the colors of the thalamic nuclei are symmetric between hemispheres but each nucleus has a unique label code and structure caption to distinguish between left and right hemisphere. This file, together with the maximum likelihood atlas, can be opened with one of the main visualization tools available (such as tkmedit, freeview or 3D-Slicer) in order to color regions when showing labels. This color lookup table can be also edited for customizing the colors or working with additional regions.

The data record derived from this work is available through Zenodo (Data Citation 2).

### Original datasets used

The used HCP data is provided by the Human Connectome Project, WU-Minn Consortium (Principal Investigators: David Van Essen and Kamil Ugurbil; 1U54MH091657) funded by the 16 NIH Institutes and Centers that support the NIH Blueprint for Neuroscience Research; and by the McDonnell Center for Systems Neuroscience at Washington University. It is available at https://db.humanconnectome.org, while the IDs of the used subjects, their approximative age and gender is given in a text file stored in the same Zenodo repository as the atlas maps (Data Citation 2).

Moreover, data from five healthy subjects taking part of the dataset explored for technical validation is available on Zenodo together with the previously mentioned custom code for thalamic parcellation (Data Citation 1).

## Technical Validation

As first validation, all images and results were visually inspected. We observed good quality of the applied image-registrations and robustness of the clustering in terms of their spatial distribution, with exception of the three previously mentioned cases where the ODF-based clustering failed to provide the expected parcellation.

### Comparison with histological atlas

**Figure 3:**
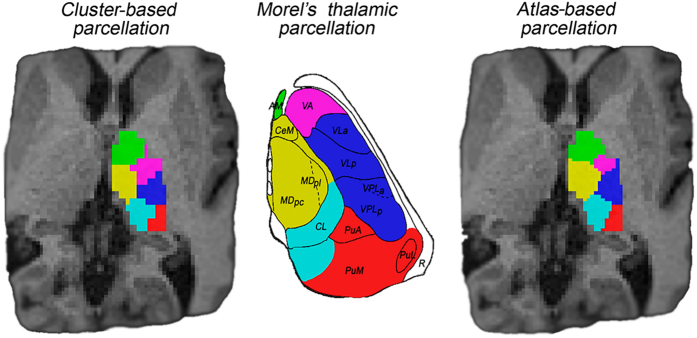
Single subject thalamic nuclei segmented with both cluster-based and atlas-based parcellation approaches.

The lack of a gold standard for the thalamic nuclei makes the assessment of the atlas-based parcellation difficult to solve. However, following the previously reported analysis by Battistella *et al.*^11^, the results obtained from the atlas-based segmentation of the thalamic subparts in subject-space were visually compared to the histological atlas of Morel. This inspection demonstrated similarity between the obtained segmentation pattern and the thalamic anatomy presented in Morel’s atlas (please refer to Fig. 3).

### Comparison with subject-based DW-MRI clustering

In order to characterize the differences that could appear when segmenting the thalamus nuclei using an atlas-based segmentation approach compared to a cluster-based parcellation using the diffusion-weighted images^11^, we analyzed an independent large cohort of additional healthy subjects.

#### Testing Dataset

The testing subset consisted of 34 healthy subjects with no history of neurological illnesses, aged between 20 and 70 years, that were scanned in a 3 T Siemens Trio scanner (Siemens AG, Erlangen, Germany) using a 32-channel head coil. Differently from the HCP, this dataset was acquired in a standard clinical setting. Local institutional review board named *Commission cantonale d'éthique de la recherche sur l'être humaine (CER-VD)* approved the study and all participants gave written informed consent. All the presented analyses were performed in accordance with the relevent ethical ragulations.

***T1w***: The Gradient Echo (MPRAGE) images were acquired with echo time (TE) = 2.98 ms, repetition time (TR) = 2300 ms, flip angle (FA) = 8^o^, field of view (FOV) = 160 × 256 × 256 mm^3^, voxel size = 1 × 1 × 1 mm^3^ and acquisition time 4 min 4 s.

***DW-MRI***: DW-MRI were acquired using a spin-echo echo-planar imaging sequence with echo time (TE) = 89 ms, repetition time (TR) = 6700 ms, field of view (FOV) = 192 × 192 × 130 mm^3^, voxel size = 2 × 2 × 2.5 mm^3^ and b-value = 1000 s/mm^2^. The gradient table includes 64 diffusion-weighted directions plus 1 b0 acquisition. The acquisition time was 8 min.

For each subject, both thalami were segmented using both approaches: atlas-based and cluster-based segmentation, making use of the T1w and diffusion-weighted images, respectively. The parcellation results provided from both methods in a single subject are shown in Figure 3. In terms of spatial distribution and extent of the delineated clusters, we visually observe similar segmentation pattern from both approaches (see Fig. 3).

#### Evaluation metrics

Three main quantitative metrics were used to evaluate the similarity between both segmentation approaches, the atlas-based segmentation ($P_{\mathrm{atlas}}$) and the cluster-based ($P_{\mathrm{clust}}$). More precisely we computed: 1) the Dice Similarity Index^32^, which measures the spatial overlap between both segmentations, 2) normalized difference in volume with respect to the volume obtained using the cluster-based approach and 3) the distance between thalamic nuclei centroids.

The centroids coordinates were computed by averaging the coordinates of the voxels that belong to the same thalamic nucleus. For each thalamic nucleus and parcellation method, the distance between the centroid and the farthest point on the nucleus was also computed. This metric can be considered as a measure of the radius of each thalamic nuclei and is used as a reference value for evaluating the magnitude of the distance between the nuclei centroids obtained from both parcellation methods.

The Dice coefficient is one of the measures of the extent of spatial overlap between two binary images. It is commonly used in reporting segmentation performance and gives more weighting to instances where the two images agree. Its values range between 0 (no overlap) and 1 (perfect agreement). In this work, the Dice values are obtained using the following expression (1)]:

(1)Dice(Patlas,Pclust)=2(Patlas∩Pclust)(Patlas∩Pclust+Patlas∪Pclust)

Percentage of volume difference between both parcellations relative to volume obtained by using the cluster-based approach was calculated as follow:

(2)%ΔV=Vatlas−VclustVclust×100

**Figure 4:**
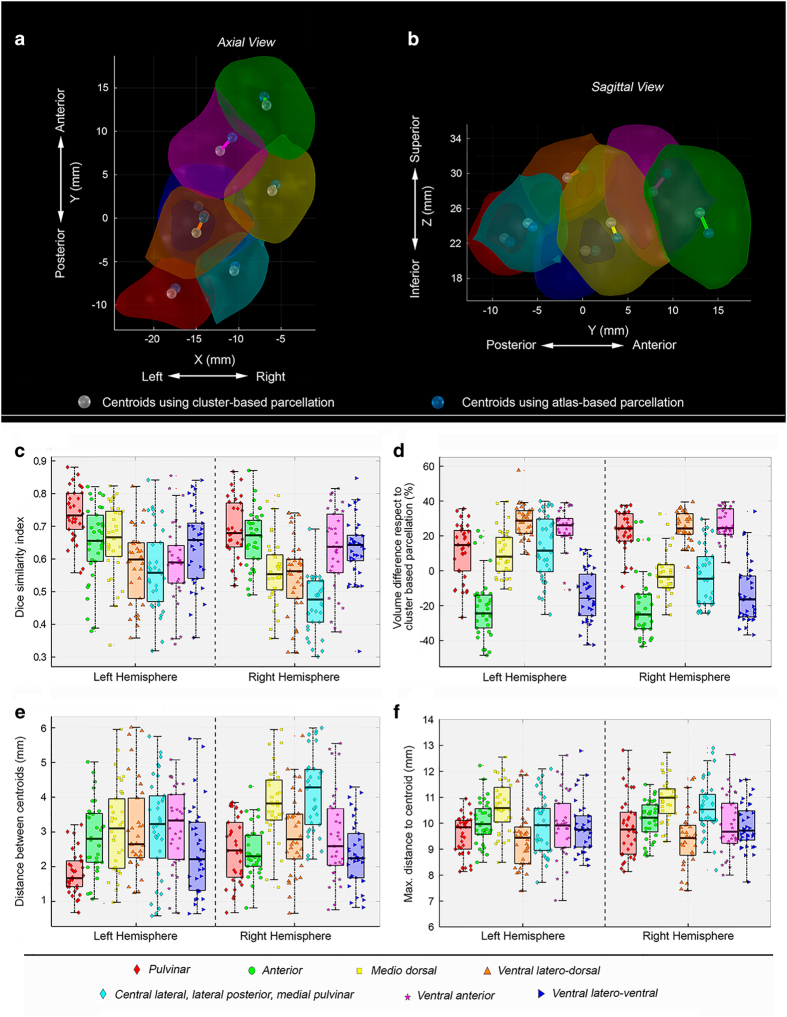
Different similarity metrics between both cluster-based and atlas-based thalamic parcellation methods for each subject and each thalamus. (**a**) Axial view and (**b**) Sagittal view of the cluster-based segmentation of the right thalamus in a single subject. The centroids obtained from both methodologies, the cluster-based and the atlas-based, are given in white and blue respectively. (**c**) Dice similarity index. (**d**) Relative percentage of volume differences. (**e**) Distance between the centroids coordinates. The metrics shown in (**c**-**e**) were calculated between both approaches. (**f**) Maximum radius of each nuclei obtained by the cluster-based parcellation. This value serves as a reference for evaluating the distance between the centroids of the nuclei obtained by both methods. For each boxplot, the median values are shown together with the confidence interval (25% and the 75% percentiles).

Figure 4a and b show the corresponding surfaces of the cluster-based parcellation for a selected subject and the centroids obtained from both segmentation approaches, atlas-based and cluster-based, respectively. An overview of all the results is given in Table 2 and Fig. 4.

The Dice Similarity Index (Fig. 4c) showed an overlap of 0.6 on average, where the maximum was found for the pulvinar left (0.74) and the minimum for the Cl-LP-PuM right (0.46). Regarding the size of the clusters, these findings could be considered as a fair match. Complementary to Dice, the volume difference indicates that overall there is only 20% of size-mismatch between the clusters obtained from both methods (see Fig. 4d). Additionally, the Euclidean distance between center of mass of atlas-based segmentation and those of the direct clustering-based segmentation reflect also an important degree of agreement in terms of locations (Fig. 4e). Pulvinar and ventral latero-ventral show low values of distance between centroids, while the maximal variation appears for the cluster CL-LP-PuM in the right hemisphere. Nevertheless, from the reported results we see that the average centroids difference for this cluster is three times less than the maximal radius of the cluster (see Fig. 4c and f). For the others clusters this distance corresponds to even smaller proportion of the estimated radius. Hence, the variation between the centers of mass of the respective clusters obtained from the two approaches can be considered as relatively low.

## Additional information

**How to cite this article**: Najdenovska, E. *et al.* *In-vivo* probabilistic atlas of human thalamic nuclei based on diffusion-weighted magnetic resonance imaging. *Sci. Data*. 5:180270 doi: 10.1038/sdata.2018.270 (2018).

**Publisher’s note**: Springer Nature remains neutral with regard to jurisdictional claims in published maps and institutional affiliations.

## Supplementary Material



## Figures and Tables

**Table 1:** Summary of the data records related to this study.

Dataset	Human Connectome Project (HCP) cohort	Testing cohort
Number of subjects	70	34
Provenance	refer to https://db.humanconnectome.org	control group of the study presented by Battistella *et al.*^31^
Available from	https://db.humanconnectome.org	a subset available in Data Citation 1
Used modalities	T1w and DW-MRI	T1w and DW-MRI
Experimental usage	Building the atlas	Technical validation of the atlas
Provided output	Thalamic nuclei SPAMs and maximum likelihood atlas	Atlas-based and cluster-based thalamic parcellation
Output format	NifTI-1 (extension .nii.gz)	NifTI-1 (extension .nii.gz)

**Table 2:** Similarity metrics between both thalamic parcellation algorithms.

Thalamus Nuclei	Dice	Percentage of volume difference *(%)*	Distance between centroids *(mm)*	Maximum distance to centroid *(mm)*
Left Hemisphere				
*Pulvinar*	0.74±0.08	11.45±15.94	1.80±0.59	8.99±0.76
*Anterior*	0.65±0.12	-21.44±17.49	2.85±1.01	8.19±0.88
*Medio-dorsal*	0.66±0.11	0.10±13.25	3.08±1.23	8.78±0.62
*Ventral latero-dorsal*	0.58±0.11	28.29±9.55	3.08±1.31	10.12±0.67
CL-LP-PuM*	0.57±0.12	13.26±18.30	3.14±1.42	10.14±0.90
*Ventral anterior*	0.59±0.12	24.45±10.83	3.13±1.12	9.10±0.61
*Ventral latero-ventral*	0.64±0.12	−14.30±15.11	2.43±1.28	8.49±0.77
Right Hemisphere				
*Pulvinar*	0.69±0.09	22.69±11.47	2.48±0.93	9.24±0.58
*Anterior*	0.66±0.09	−19.76±19.33	2.41±0.70	8.60±0.71
*Medio-dorsal*	0.56±0.10	−2.17±12.37	3.81±1.02	8.67±0.64
*Ventral latero-dorsal*	0.55±0.12	24.62±8.82	2.93±1.14	9.99±0.73
CL-LP-PuM*	0.46±0.09	−3.58±16.99	4.12±1.09	8.60±0.65
*Ventral anterior*	0.63±0.12	26.70±8.61	2.84±1.26	9.69±0.57
*Ventral latero-ventral*	0.63±0.10	−13.14±17.09	2.36±0.94	8.64±0.79
*Central lateral, lateral posterior, medial pulvinar.				
